# Editorial: Treatment of subclinical thyroid dysfunction in patients with comorbidities

**DOI:** 10.3389/fendo.2023.1141612

**Published:** 2023-01-20

**Authors:** Salman Razvi, Leonidas Duntas, Bernadette Biondi

**Affiliations:** ^1^ Translational and Clinical Research Institute, Newcastle University, Newcastle upon Tyne, United Kingdom; ^2^ Evgenideion Hospital, Unit of Endocrinology, Metabolism and Diabetes, University of Athens, Athens, Greece; ^3^ Department of Clinical Medicine and Surgery, Federico II University of Naples, Naples, Italy

**Keywords:** subclinical hyper- and hypothyroidism, levothyroxine, pregnancy, heart failure, hospital in-patients, older person, non-thyroidal illness

Thyroid hormones (TH) play a critical role in regulating growth and metabolism in almost all tissues (1). Overt thyroid diseases, when TH are clearly abnormal, lead to several cardiovascular complications and osteoporosis. Subclinical thyroid dysfunction (SCTD), a condition where TH are within the normal reference range while serum thyrotropin (TSH) is not, is more common than overt thyroid disease. However, it remains unclear what TSH cut-off value is associated with adverse outcomes in patients with SCTD; consequently, treatment of this condition remains controversial. The main issues that are yet to be elucidated pertain to definition (what constitutes abnormal thyroid function: TSH alone or TSH/TH in conjunction), treatment (who, when and how to treat), and long-term management (when to recheck and target TSH and/or TH levels to aim for). In this topic collection, five articles addressed the effects of STD respectively in pregnancy, older patients, and patients with comorbidities (hospitalized patients, patients with heart failure and patients with COVID-19).

The management of hypothyroidism in hospitalised patients has received little attention in the scientific literature. It is well recognised that almost half of patients on levothyroxine treatment are inadequately treated and both over-and under-treatment of hypothyroidism are associated with adverse outcomes (2). In a multicentre cluster randomised trial comparing a standardised educational programme versus no programme, clinicians from Italy found that education significantly improved some indicators of hypothyroidism management (Brancato et al.). This suggests that more structured education of clinicians managing hospitalised patients with hypothyroidism is required. Further studies are needed to evaluate if clinician education is clinically beneficial and is cost-effective.

The management of subclinical hypothyroidism (SCH) in pregnancy remains a controversial issue. This is more so as the associated risks are not just for the mother but also for the offspring. In particular, the TSH diagnostic cut-off used to define SCH has been contentious (3, 4). The American Thyroid Association (ATA) strongly recommends replacement therapy with levothyroxine when serum TSH is >10.0 mU/L or when serum TSH >4.0 mU/L or 2.5 mU/L (low to moderate quality evidence according to TPOAb status and individual patient circumstances) (3). In a systematic review and meta-analysis of 6 studies that included nearly 8000 participants and defined SCH as serum TSH >4.0 mU/L in the first trimester, researchers found that levothyroxine treatment of SCH in pregnant women was associated with a 45% reduced risk of pregnancy loss, 37% lower risk of preterm birth, and a 22% lower risk of pregnancy-induced hypertension. In a subgroup analysis, there was no significant interaction of the benefits of treatment based on TPOAb status (Ding et al.).

There is a modest rise in serum TSH levels with age, yet the TSH reference range does not reflect this age-related increase (5). It is therefore not surprising that the prevalence of SCH rises with age and there is an attenuation of this increase when age-appropriate TSH reference ranges are utilised (6). However, randomised controlled trials of levothyroxine treatment of SCH in older people (>65 years) conducted to date have been proven to be ineffective in improving symptoms of hypothyroidism and/or quality of life (7). Yet, it remains unclear whether other health-related parameters may be impacted by treatment. In a systematic review and meta-analysis, Zhao et al. have assessed the effects of levothyroxine in 13 trials in 5000 older people with SCH. They conclude that levothyroxine treatment may reduce lipid-related parameters (such as total and LDL-cholesterol, triglycerides and apolipoprotein B but has no impact on symptoms, body weight, blood pressure, cognitive function or symptoms of depression.

The cardiovascular system in general and the myocardium in particular are important targets of TH action (8). A number of observational studies have demonstrated that SCH is associated with worse outcomes in patients with heart failure. However, there have been very few interventional trials that included a small number of participants that have assessed the impact of levothyroxine treatment on myocardial function in chronic heart failure patients with SCH (8). Trigiani and colleagues have performed a systematic review of two clinical trials and concluded that the current evidence is inadequate to support levothyroxine treatment of SCH in heart failure patients and that adequately designed and dose-response trials are required (Triggiani et al.).

TH are impacted by illness and the prevalence of thyroid dysfunction related to non-thyroidal illness (NTI) depends on the population and the severity of the illness (9). In a systematic review and meta-analysis, Darvishi et al. have assessed the prevalence of thyroid dysfunction in patients with COVID-19 and whether thyroid dysfunction predicted outcomes. Amongst 30 studies that included nearly 10000 patients with COVID-19, the overall prevalence of thyroid dysfunction was 15%, ranging between 6.2% in those with mild to moderate disease and 20.8% in those with severe to critical illness. The level of TSH however did not predict mortality, however. These results once again confirm that NTI is related to the severity of the illness and that treatment may not be beneficial (Sciacchitano et al.).

In conclusion, SCTD remains common across multiple populations and across a range of conditions. Based on current evidence, three things can be concluded. First, it remains the case that pregnant women with SCH (serum TSH >4.0 mU/L) in the first trimester should be offered treatment to reduce the risk of pregnancy-related adverse outcomes. Second, older people (>65 years) with mild SCH (TSH level between 4.0 and 10.0 mU/L) should not routinely be offered treatment, particularly with the aim to improve symptoms, quality of life or body weight. Third, high quality trials of benefits and risks of treatment of SCH are required in individuals with heart failure and SCH. And, fourth, educational programmes for clinicians dealing with patients with hypothyroidism may be beneficial but long-term effects remain to be evaluated.

## Author contributions

All authors listed have made a substantial, direct, and intellectual contribution to the work and approved it for publication.
